# Understanding Occlusion and Temporomandibular Joint Function Using Deep Learning and Predictive Modeling

**DOI:** 10.1002/cre2.70028

**Published:** 2024-11-19

**Authors:** Taseef Hasan Farook, James Dudley

**Affiliations:** ^1^ Adelaide Dental School The University of Adelaide South Australia Australia

**Keywords:** chairside diagnostics, jaw movement, machine learning, mastication, parafunctional habits

## Abstract

**Objectives:**

Advancements in artificial intelligence (AI)‐driven predictive modeling in dentistry are outpacing the clinical translation of research findings. Predictive modeling uses statistical methods to anticipate norms related to TMJ dynamics, complementing imaging modalities like cone beam computed tomography (CBCT) and magnetic resonance imaging (MRI). Deep learning, a subset of AI, helps quantify and analyze complex hierarchical relationships in occlusion and TMJ function. This narrative review explores the application of predictive modeling and deep learning to identify clinical trends and associations related to occlusion and TMJ function.

**Results:**

Debates persist regarding best practices for managing occlusal factors in temporomandibular joint (TMJ) function analysis while interpreting and quantifying findings related to the TMJ and occlusion and mitigating biases remain challenging. Data generated from noninvasive chairside tools such as jaw trackers, video tracking, and 3D scanners with virtual articulators offer unique insights by predicting variations in dynamic jaw movement, TMJ, and occlusion. The predictions help us understand the highly individualized norms surrounding TMJ function that are often required to address temporomandibular disorders (TMDs) in general practice.

**Conclusions:**

Normal TMJ function, occlusion, and the appropriate management of TMDs are complex and continue to attract ongoing debate. This review examines how predictive modeling and artificial intelligence aid in understanding occlusion and TMJ function and provides insights into complex dental conditions such as TMDs that may improve diagnosis and treatment outcomes with noninvasive techniques.

## 1 Introduction

The temporomandibular joint (TMJ) connects the mandible to the temporal bone of the skull, facilitating essential actions such as chewing, speaking, and yawning (Lobbezoo et al. 2004). Occlusion refers to how the upper and lower teeth fit together when the jaws close (McLean 1938). Both TMJ function and occlusion are key considerations in temporomandibular joint disorders (TMDs) and malocclusion (Sigaroudi and Knap 1983; Hirsch et al. 2005). However, debates persist regarding optimal diagnostic methods, treatment strategies, and the significance of occlusal factors in TMD (Olliver et al. 2020; Stegenga 2010). Understanding TMJ function is challenging due to its complex composition and the diverse range of TMD symptoms which often overlap with other dental and non‐dental conditions (Stegenga 2010). Healthcare professionals lack consensus on TMD etiology and management which further complicates its comprehension (Farook and Dudley 2023a). Traditionally, assessing TMJ function involved invasive or radiation‐exposing procedures (Krishnamoorthy, Mamatha, and Kumar 2013). However, recent noninvasive or minimally invasive techniques offer promising avenues for predicting masticatory muscle activity and jaw anomalies such as inflammatory or degenerative disorders (Shoukri et al. 2019). This narrative review explores the application of artificial intelligence (AI), specifically predictive modeling, deep learning, and computer vision, in clinical dentistry using noninvasive approaches, with a focus on trends related to occlusion and TMJ function.

### 1.1 Progressive Digitization of Mandibular Functional Analyses

Medical imaging modalities such as CBCT and MRI scans continue to be the standard means for diagnosing TMD. Due to challenges associated with accessibility, ethical considerations, and administrative complexities, alternative noninvasive methods for diagnosing TMDs have been developed (Farook and Dudley 2023a; Farook, Rashid, Alam, and Dudley 2022). In the 1990s, a notable trend emerged that steered clinical research toward a computerized approach for managing TMD cases (Hayashi et al. 1994). In 1994, initial attempts at automated jaw movement quantification faced challenges with metal components, leading to inaccuracies due to weighted movements and frictional resistance (Hayashi et al. 1994). Electronic sensors were accurate to 150 microns and clinical implementation values were accurate to 330 microns; however, there were large influences from mandibular deflection, tooth articulation, and deviation (Hayashi et al. 1994). The challenges included electrical noise, Light‐emitting diode (LED) tracking affected by skull movement, and intraoral fixation impacting labial movement and anterior guidance.

Advancements in 2009 addressed head movements with lightweight prostheses and improved sensor accuracy to a 70‐micron standard deviation in mandibular motion during chewing (Röhrle et al. 2009). Subsequent developments included simulations of motion tracking on cadavers in 2011, (Celebi et al. 2011) computerized algorithms and virtual articulators predicting jaw motion from CBCT scans in 2018 (Lucena et al. 2018; Lepidi et al. 2021); since 2020, deep learning applications predicted movements instead of reading them. In 2021, intraoral sensors such as 3D scanners for motion diagnostics were in the early stages of development (Lepidi et al. 2021; O'Hare et al. 2021). By 2023, 3D intraoral scanners were capable of monitoring dynamic jaw movement functions, with the latest devices combining camera tracking, wearable sensors, and predictive modeling achieving accuracies of 50–100 microns (Farook, Rashid, Alam, and Dudley 2022). By 2024, predictive modeling and deep learning have taken a generational leap in tracking dynamic jaw movements and identifying clinical trends influencing occlusion and TMJ function (Kois, Zeitler, and Revilla‐León 2024).

### 1.2 Limitations of Traditional Functional Analyses

Therapeutic Goods Administration (TGA)‐approved electronic devices have limitations and controversies. Manual facebow articulators involve mounting models on an articulator or using digital or wearable devices to replicate jaw movements, which are susceptible to subjective biases (Lepidi et al. 2021). Axiography faces challenges with a heavy, technique‐sensitive recording apparatus, requiring intraoral stabilization (Farook, Rashid, Alam, and Dudley 2022). Technologies such as axiography, electromyography, electrognathography, virtual articulators, radiomics‐guided jaw tracking, and optoelectronic tracking offer diverse approaches, yet high costs, and the need for subjective interpretation limit access (Farook, Rashid, Alam, and Dudley 2022). Intraoral stabilization controversies arise from inhibiting natural jaw movements (Farook, Ahmed, Talukder, and Dudley 2023). Digital jaw movement tracing using optoelectronic sensors (Grande et al. 2024), 3D scanning, ultrasonic signals, radiographic imaging, and photogrammetry highlight possibilities but face challenges such as environmental noise, variations in clinical implementations, device processing capabilities, and low inter‐rater reliability (Farook, Rashid, Alam, and Dudley 2022; Revilla‐León et al. 2020). Plotting landmarks from photographs and video recording is advocated as a more accessible approach, acknowledging issues with operator‐labeled images being prone to observer biases (Rousseau and Retrouvey 2022).

### 1.3 The Lack of Established Norms

Many specialized chairside devices, such as the axiography, electrognathograph, electromyograph, occlusal pressure sensors, etc, have been developed over time that provide unique information on the TMJ and occlusion (Farook, Ahmed, Talukder, and Dudley 2023). Yet, autonomously combining and quantifying this information while simultaneously neutralizing sources of biases and errors has historically been a challenge. The standardized capacity of complex jaw movement is uncertain, and establishing norms poses significant challenges, where predictive modeling can help establish some much‐needed norms without challenging the validity of medical imaging modalities such as CBCT and MRI scans.

## 2 Making Predictions

Predictive modeling uses statistical methods to anticipate unknown outcomes (Steyerberg and Steyerberg 2009). The term “model” in predictive modeling denotes a mathematical or computational depiction of the connection between predictor variables and an outcome variable, aiming to use historical data for precise predictions about future or unseen data (Alanazi et al. 2018). Historically in medical AI, two prominent categories of predictive modeling have been supervised and semi‐supervised learning (Farook and Dudley 2023a; Pethani 2021). Supervised models require humans to actively classify regions of interest, while semi/weakly supervised learning enables AI models to learn from data with limited human interactions with the data sets (Berry, Mohamed, and Yap 2019).

### 2.1 The Role of Deep Learning

**Figure 1:**
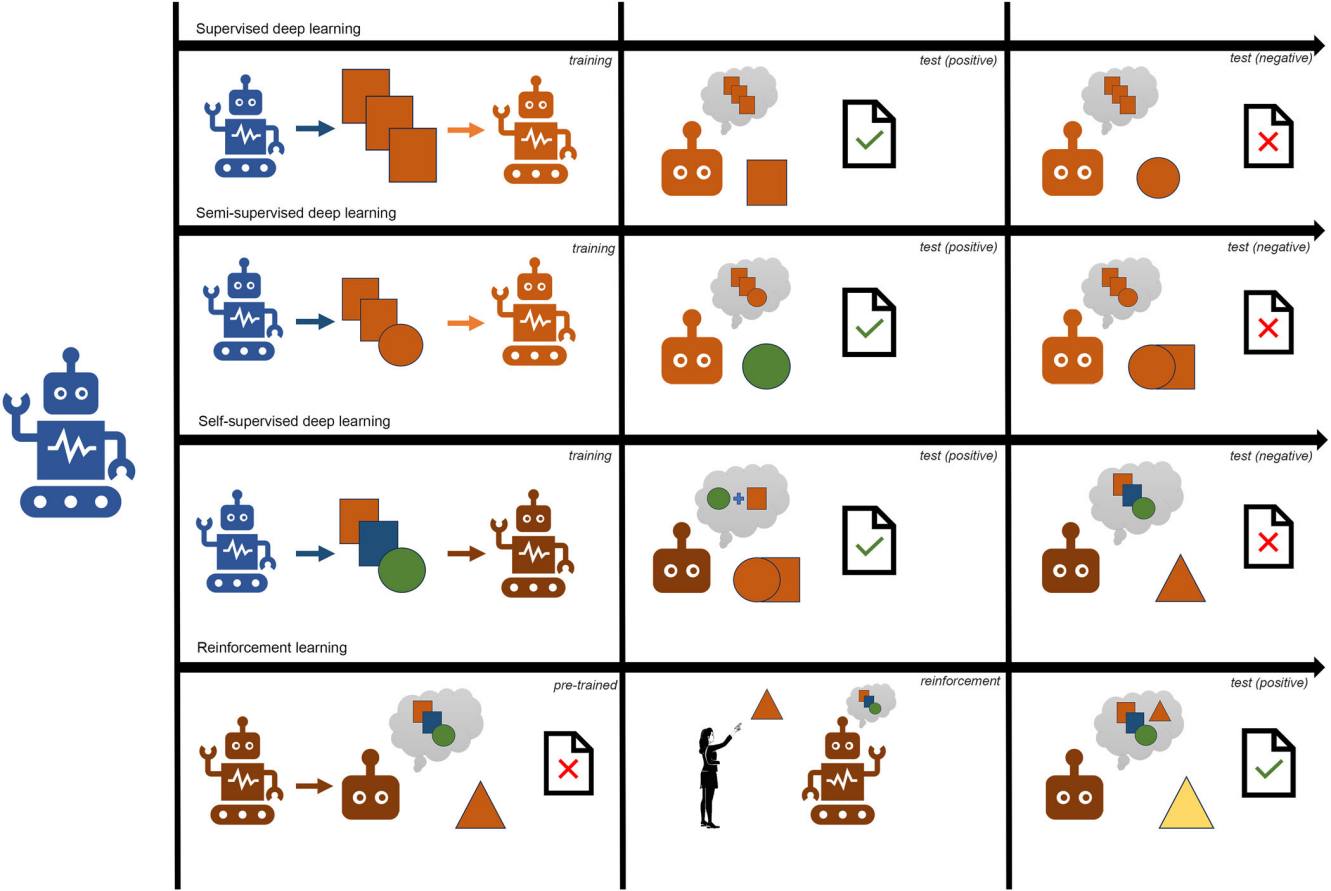
A simplified illustration showcasing the capabilities and limitations of various types of deep learning models.

When complex hierarchical relationships are analyzed, deep learning is applied which is a concept related to predictive modeling and within the umbrella of AI (Mohammad‐Rahimi et al. 2022). Medical imaging data are often processed through a deep learning process known as computer vision with a focus on interpreting visual information. Traditional supervised medical deep learning follows a process where an AI model is taught to distinguish between two or more classes of data variations after evaluating a series of interrelated data. Subsequently, the model's accuracy is tested in identifying and predicting classes when presented with similar but previously unseen data, a task it has not encountered during the training phase. More recently, there has been a shift toward self‐supervised learning, where a pretrained model can generate results without the need for constant training or human interaction (Farook, Ahmed, Giri, Rashid, Hughes, and Dudley 2023). Such models are prone to making mistakes, that are corrected by human operators in subsequent iterations through the process of reinforcement learning (Kaelbling, Littman, and Moore 1995). Figure 1 illustrates various forms of deep learning models currently used in dentistry and medical research, providing a simplified representation of how accurate predictions are made for easy understanding.

### 2.2 The Impact of Data Acquisition Techniques on Predictions

Predictive modeling exhibits inherent imperfections based on the quality of the data. There is a substantial influence of clinic surroundings and ambient light exposure on real‐time fiducial marker tracking (Saad et al. 2024). The effectiveness of marker tracking is further affected by video compression methods and subtle habitual lateral head movements (Saad et al. 2024). The challenges encountered in automated diagnostics and predictive modeling of TMJ function through AI are intricately connected to the processing quality of medical imaging data sets (Farook and Dudley 2023a). Instances of suboptimal exposures in CT imaging or procedural errors in the cephalometric landmark and occlusal analyses introduce subjective variables posing hurdles to standardization. Additionally, device‐influenced variables, including imaging quality, impede automation and deep learning analyses in this complex domain.

### 2.3 Data Manipulation

**Figure 2:**
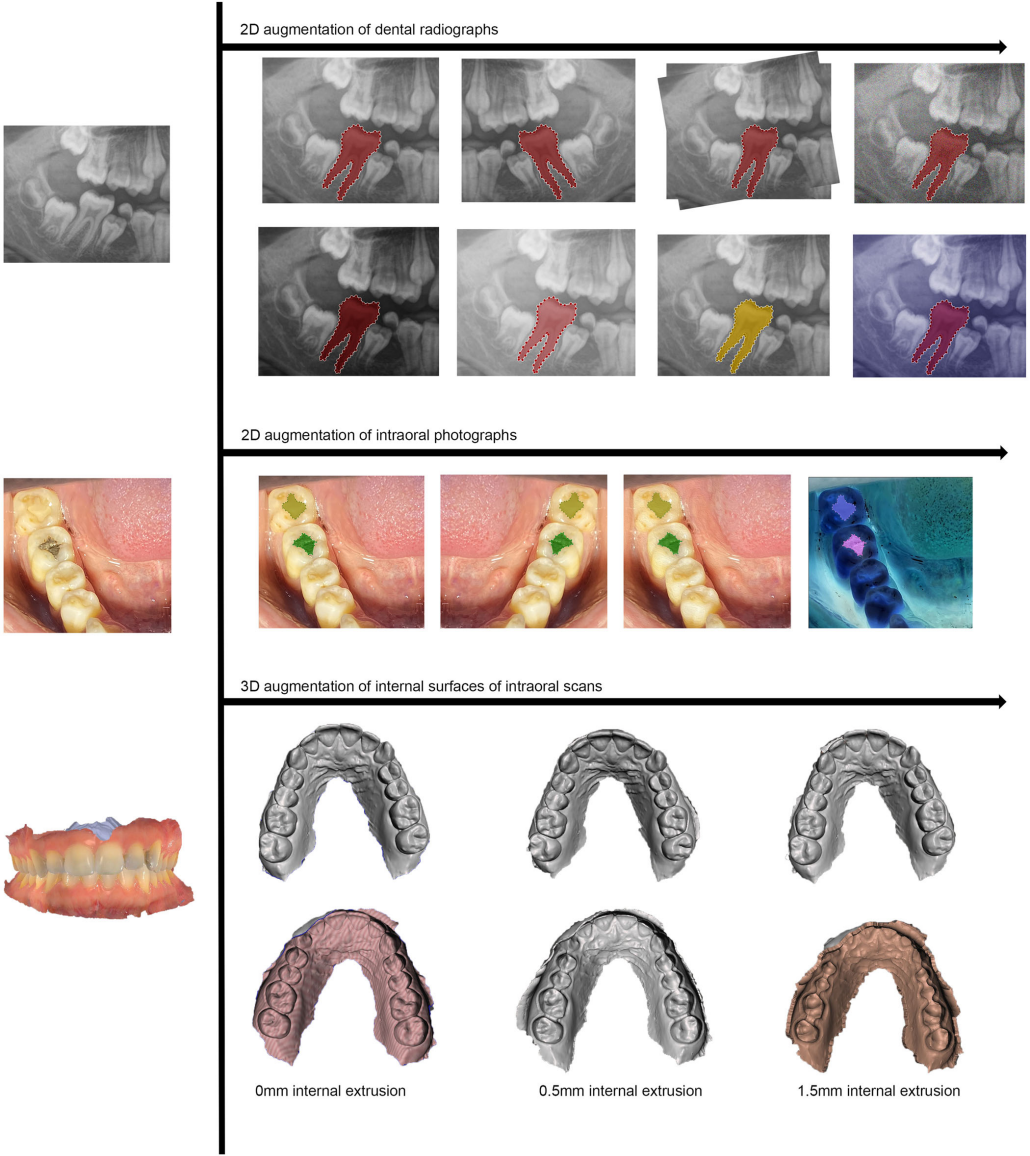
Data augmentation process applied to labeled dental radiographs, intraoral photographs, and 3D scans to introduce variations into a clinical data set before deep learning analysis.

Exploratory data analysis (EDA) in medical data sciences serves as an important step to understanding research data, discovering patterns, and predicting relationships and anomalies (Milo and Somech 2020). A meaningful EDA requires very large volumes of data, which is often inaccessible owing to privacy or ethical constraints. Data augmentation, a method of generating new data by manipulating certain patterns in the original data set, has been a common practice in radiology to train medical deep learning models to compensate for the lack of extensive data sets (Chlap et al. 2021; Van Dyk and Meng 2001). In dental research, different augmentation methods have been applied to introduce variations to intraoral images and radiographs (Hasan et al. 2023; Farook, Saad, Ahmed, Dudley, and Saad 2023). While skepticism may surround the authenticity of augmented data, recent advances have surpassed the augmentation of 2D data and researchers have begun to identify appropriate methods to augment 3D intraoral scans (Farook, Ahmed, Giri, Rashid, Hughes, and Dudley 2023). A simple yet significant method involves adding layers to internal surfaces, ensuring minute volumetric change without modifying external details present on the tooth surface (Farook, Ahmed, Giri, Rashid, Hughes, and Dudley 2023). Figure 2 highlights several results of data augmentation, with the potential for augmentation extending beyond the examples depicted in the figure.

### 2.4 Augmenting 3D Intraoral Scans

**Figure 3:**
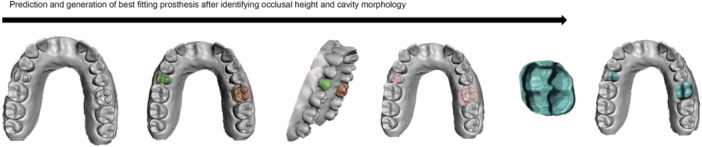
A workflow to facilitate the generation of dental prostheses based on predictive modeling of properties related to cavity morphology and occlusion.

It has been observed that choices of scanner and operator had no significant impact on the accuracy of AI predictions (Farook, Ahmed, Giri, Rashid, Hughes, and Dudley 2023). However, the augmentation procedure enabled the utilization of standard intraoral scan data in unsupervised deep learning, showcasing the potential for any standard clinical practice with access to an intraoral scanner to contribute 3D models to deduce optimal occlusal parameters and construct prostheses automatically. Figure 3 illustrates one of the initial applications of this technology to generate partial dental crowns through predictive modeling of augmented scans.

### 2.5 Benefits of Tracking Facial Movement Through Deep Learning

The historical challenges faced in real‐time tracking of facial landmarks for maxillofacial feature measurements are rooted in reliability issues stemming from operator intervention and biases (Rousseau and Retrouvey 2022). Traditional speech pathology and phono‐articulatory analyses, which concentrate on jaw movements during speech and function, generate qualitative outputs characterized by subjective biases (Hu et al. 2013). The accuracy of facial landmark tracking and speech analysis holds importance in various applications within oral and maxillofacial specialties (Everett and Chen 2021). However, this accuracy encounters obstacles due to individual heterogeneity, non‐standardized monitoring methods, operator biases, and limited generalizability (Farook, Rashid, Alam, and Dudley 2022).

The advent of automated real‐time facial parameter measurements through AI marks a significant advancement, enabling research on soft tissue morphology and the identification of habitual changes during dynamic jaw movements (King 2009; Farook, Saad, Ahmed, and Dudley 2023a). This process relies on deep learning models to identify facial landmarks, reducing the manual effort required for video analysis in dental soft tissue landmark tracking (Farook, Saad, Ahmed, and Dudley 2023a). The result is enhanced consistency and universality across variables such as gender, age, ethnicity, and facial features when determining trends in facial landmark variations (Serengil and Ozpinar 2020).

### 2.6 Overcoming Language and Cultural Barriers for Speech Assessment

Deep learning currently plays an important role in clinical audio deciphering, employing large‐scale, weakly supervised models for speech recognition (Farook, Saad, Ahmed, and Dudley 2023b). Essentially, deep learning can recognize individuals or keywords during dental office conversations, regardless of accents or dialects, extracting timestamped and transcribed speech from patients and correlating the extracted audio signals with facial landmark changes (Farook, Ramees, and Dudley 2024a). The overarching goal is to enhance comprehension of jaw movement patterns and speech impediments across language and cultural barriers (Farook, Saad, Ahmed, and Dudley 2023b).

### 2.7 Achievable Outcomes Through Predictive Modeling in TMJ Functional Analysis

**Figure 4:**
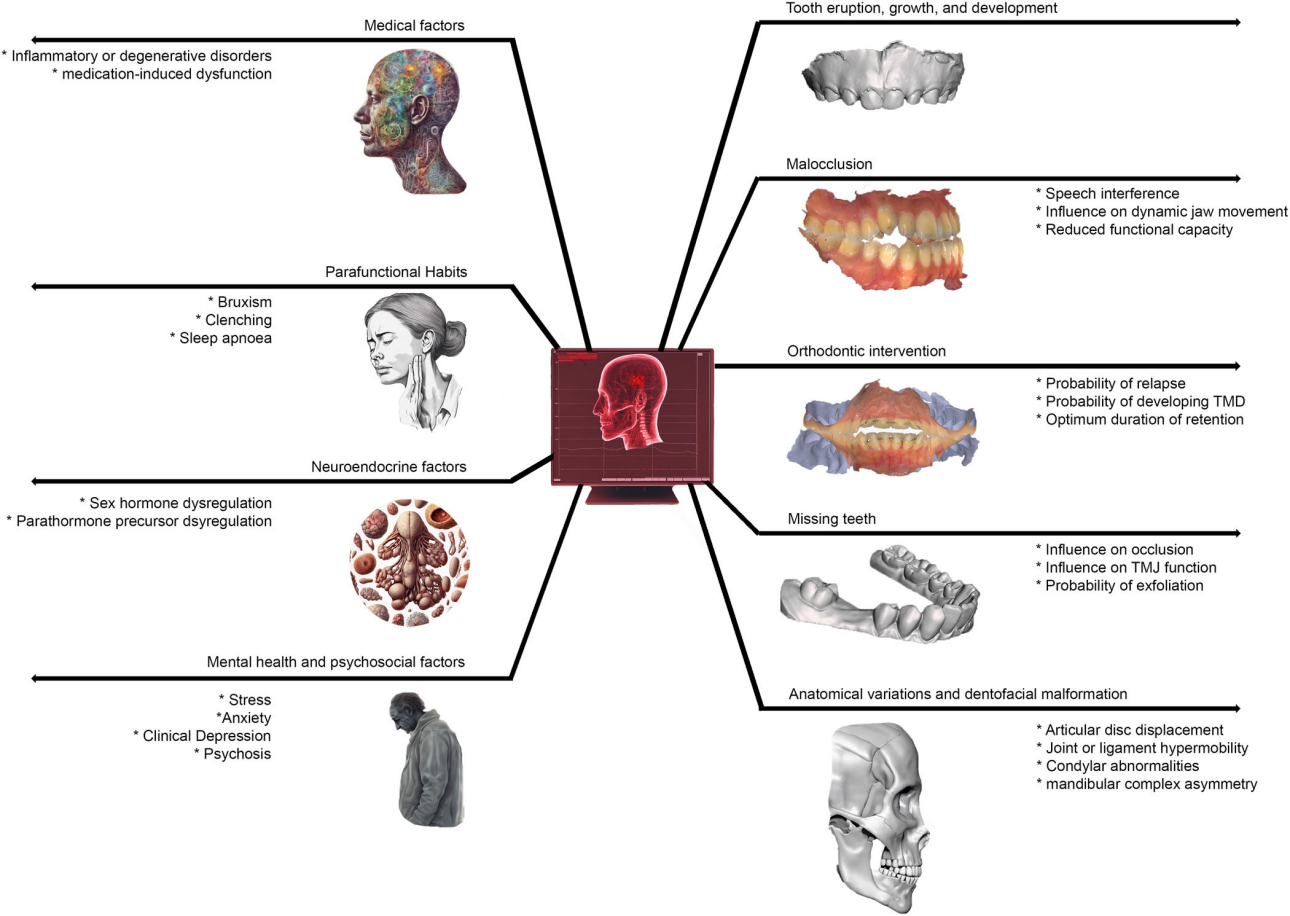
Predictions enabled by dental data alone (right) or in combination with a comprehensive medical history and investigation reports (left).

Predictive modeling can offer insights into the probability of a patient having or developing certain TMJ conditions when considering various patient‐specific variables. Figure 4 illustrates scenarios where predictive modeling may help identify factors influencing TMD that have historically posed challenges in clinics (de Araújo et al. 2014).

## 3 Predicting Occlusion

Dental occlusion is complex and challenging to standardize digitally due to the large number of influencing factors. Altered physiological states from missing teeth, skeletal variations, maxillofacial trauma, and parafunctional habits such as tooth grinding have been associated with physiologic or pathologic occlusion changes (McLean 1938; Klineberg and Eckert 2015). Subjective habits such as object sucking or tongue thrusting also affect occlusion (Farook, Rashid, Ahmed, and Dudley 2023). Malocclusion is one of the most prevalent oral diseases globally and often requires clinical intervention to prevent deterioration, unwanted occlusal changes, bone damage, and neuropathic pain (Lombardo et al. 2020). Such conditions frequently impact speech and the freeway space, the physiological gap between the jaws at rest (Widmalm et al. 2016; Savastano 2023).

### 3.1 Importance of Anatomical Landmarks

While algorithms for predictive modeling demonstrate robustness in accurate diagnostics of occlusal features for the mixed dentition, validation necessitates operator interventions with the learning curve for operators being steep. Most investigations on predicting matters relating to occlusion have relied on medical imaging and landmark detection either from radiographs or clinical photographs, presenting challenges in areas prone to soft tissue contractions and variations across resting facial expressions (Farook, Rashid, Ahmed, and Dudley 2023). In radiographic imaging, both hard and soft tissue landmarks in mobile areas such as menton and pogonion exhibited the largest prediction errors, suggesting difficulties in chin prediction (Park et al. 2021). Models struggle to differentiate between Class 1 and Class 2 skeletal relationships due to large variations in the subnasale space (Rousseau and Retrouvey 2022; Lu, Ko, and Liu 2009; Juneja et al. 2020). Dental malocclusion, affecting the soft tissue profile, showcased differences in machine learning performance based on whether photographic datasets or radiographs were used in identifying features (Farook, Rashid, Ahmed, and Dudley 2023). Studies automating cephalometric diagnostics found gnathion, antegonion, and menton to be important mandibular landmarks, while anterior nasal spine, crista galli, Z‐plane, and J‐plane were considered important maxillofacial landmarks in the prediction process (Choi et al. 2022; Niño‐Sandoval et al. 2017). Ensuring radiographs provide clear details for the said landmarks and calibrated measurements are performed, accurate detection of angular changes is possible across cephalometric planes, predicting progressive skeletal changes during treatment for occlusal disorders (Perillo et al. 2021).

### 3.2 Effective Data

Real‐time decision support systems developed from models with the aim of classifying maxillomandibular disorders and occlusal disharmony require large numbers of true positive cases within data sets. This may be challenging for uncommon clinical manifestations unless the data are combined from multiple centers, which is currently not possible with the strict governance of medical data and the absence of regulations on the usage of AI algorithms (Farook and Dudley 2023a). Research on the subject often excludes subjects with facial infections and trauma, introducing selection bias and limiting the learning capacity of the models (Shin et al. 2021). Recent simulation studies suggested that jaw deformities might not need to be excluded, emphasizing the potential of transferring models trained on specific conditions through a process called transfer learning for broader study designs (Jeong et al. 2021). The lack of consideration for abstract subjective conditions in patient data, such as psychological status, underscored a need for future investigation into their inclusion as studies indicate such parameters alongside standardized indices such as the Index of Orthodontic Treatment Need (IOTN) can help models understand individual scenarios better and improve prediction accuracy (Farook, Rashid, Ahmed, and Dudley 2023; Alvarez‐Arenal et al. 2020).

### 3.3 Longitudinal Data

Should academics and practitioners choose to participate in the development of predictive decision support systems, they should consider training models with longitudinal data that follow patients through various stages of their lives. Limited real‐time monitoring of treatment progression can be considered adequate at preoperative, immediate postoperative, and 12–15 months postoperative periods in the absence of additional resources to carry out more thorough follow‐ups (Choi et al. 2022). Age was found to not be a determinant of occlusal vertical dimension, while biological gender and facial type were likely to affect occlusal parameters indicating that the duration of follow‐up of a single type of condition may have less impact than introducing demographic variations to the condition (Farook, Rashid, Alam, and Dudley 2022). Measuring maxillomandibular motion for predictive modeling required five to six repetitions of jaw motion per patient, with additional movements producing minimal variations suggesting that very limited resources may be required to obtain the data necessary to build deep learning‐based prediction models and identify risks and trends (Calil et al. 2020).

### 3.4 Predicting Occlusal Traits Concerning Phonetics

**Figure 5:**
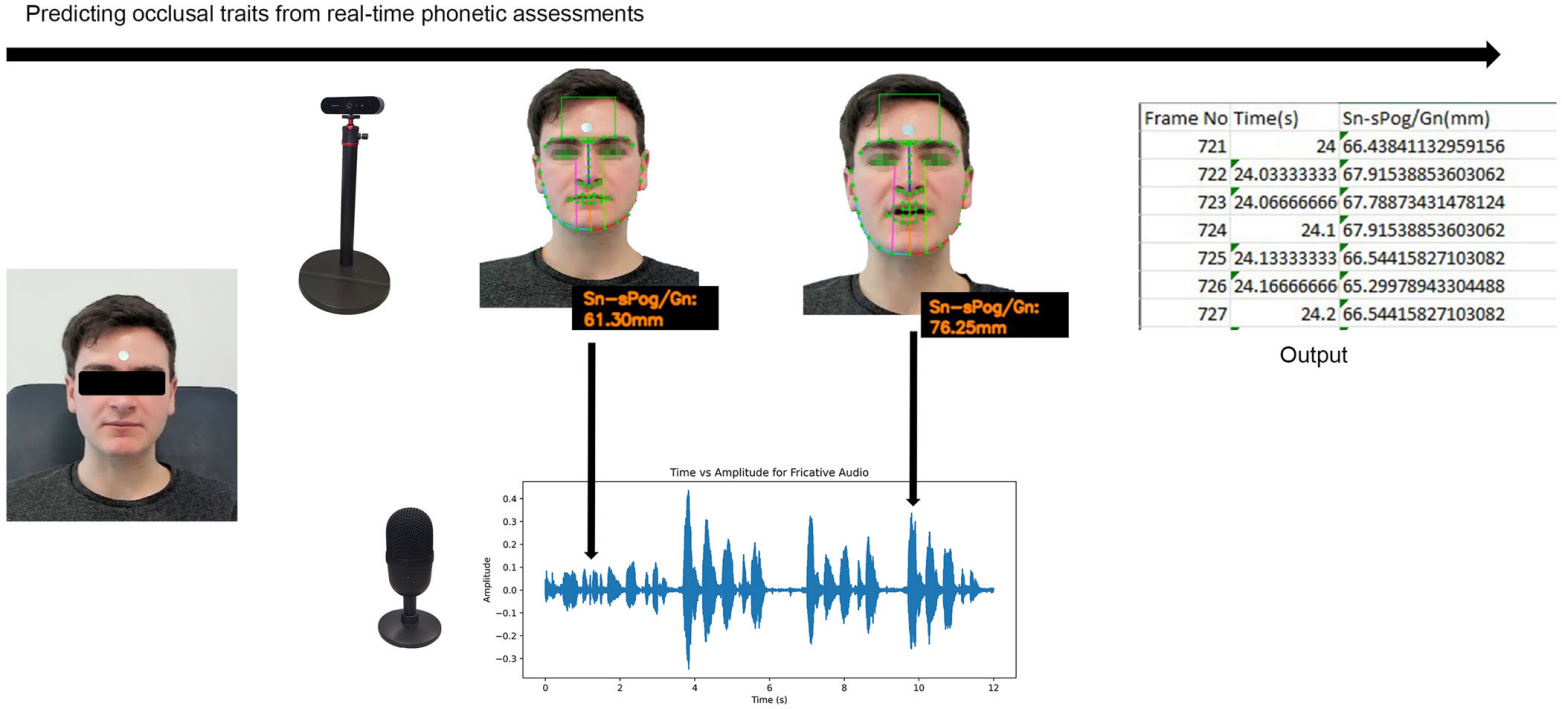
Prediction of mandibular movement patterns by establishing a relationship between real‐time mandibular displacement during speech and audio output.

Evaluating phonetics and its relationship to occlusion in dentistry is challenging due to the inherent subjectivity, variability in speech perception, and individual differences in occlusion. Achieving standardization proves difficult given the diverse speech patterns, linguistic dialects, and cultural influences present (Everett and Chen 2021). In this context, predictive modeling of soft tissue movement during phonetics can be conducted alongside evaluating hard tissue displacement using electrognathography. AI‐driven audio deciphering techniques have been shown to successfully correlate speech patterns with mandibular soft tissue displacement (Figure 5), highlighting minimal influence from dialects and pronunciation variations across ethnicities, ideal for multicultural communities (Farook, Saad, Ahmed, and Dudley 2023b).

### 3.5 Predicting Freeway Space Changes

**Figure 6:**
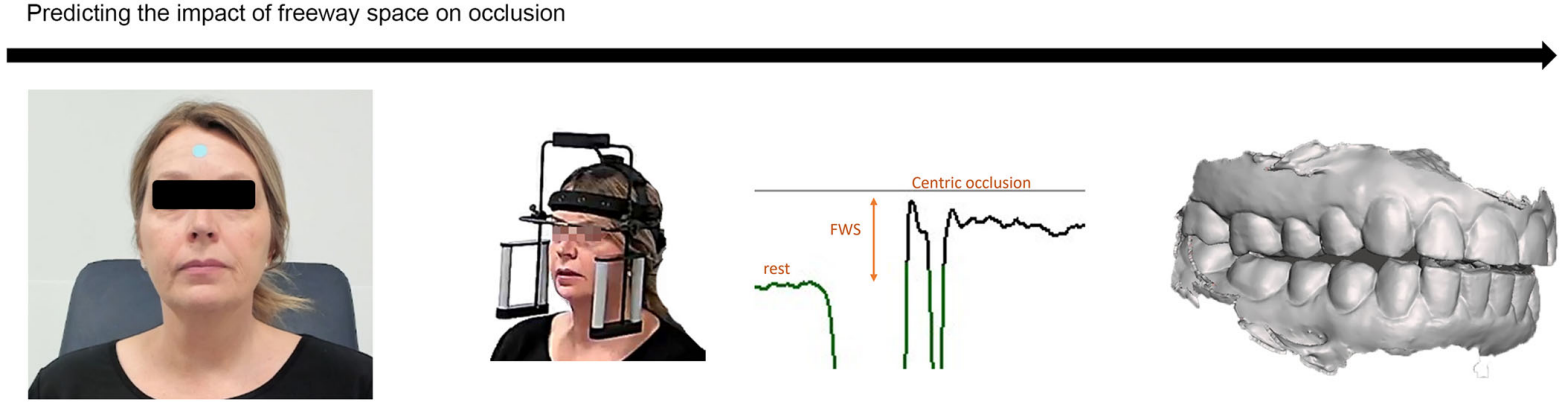
Determining the relationship between freeway space and occlusal characteristics.

Freeway space, also known as interocclusal clearance, significantly impacts the stability, comfort, and functionality of prosthetic devices, while also playing a role in preventing TMJ disorders and muscle fatigue (Savastano 2023). Deep learning‐based prediction models excel in identifying subtle deviations in jaw movement from common trends that could contribute to diminished freeway space (Figure 6). Factors such as age‐related changes, variations in height and weight, soft tissue lateral excursions, habitual head tilting, arch perimeter imbalances, and malocclusion add layers of complexity to understanding freeway space dynamics, and while this complicates diagnoses for operators, it aids models to learn of intricate variations (Halim, Sumarsongko, and Adenan 2015; Leavy, Cisneros, and LeBlanc 2016).

## 4 Predicting Temporomandibular Joint Function

TMJ rehabilitation seeks to identify the cause of the dysfunction, restore acceptable levels of joint function, and alleviate symptoms (Farook and Dudley 2023b). Clinical studies often prioritize training AI models to differentiate between positional disorders (derangement, luxation, subluxation, adherence, ankylosis) and structural disorders (congenital growth disorders, inflammatory arthritis) (Stegenga 2010; Ahmad and Schiffman 2016). While logical, experts argue that this approach may lack pragmatism, as both types often coexist and manifest similar symptoms (Stegenga 2010). Inflammatory disorders, further categorized into low and high inflammatory arthritis, face classification challenges due to limited data sets for even distribution across classifiers (Perillo et al. 2021; Ahmad and Schiffman 2016). Clinical classification complexities result in simplified binary classifications in studies, with limited access to diverse data sets due to geographic, ethical, and data protection constraints (Farook and Dudley 2023a).

### 4.1 The Black Box

**Figure 7:**
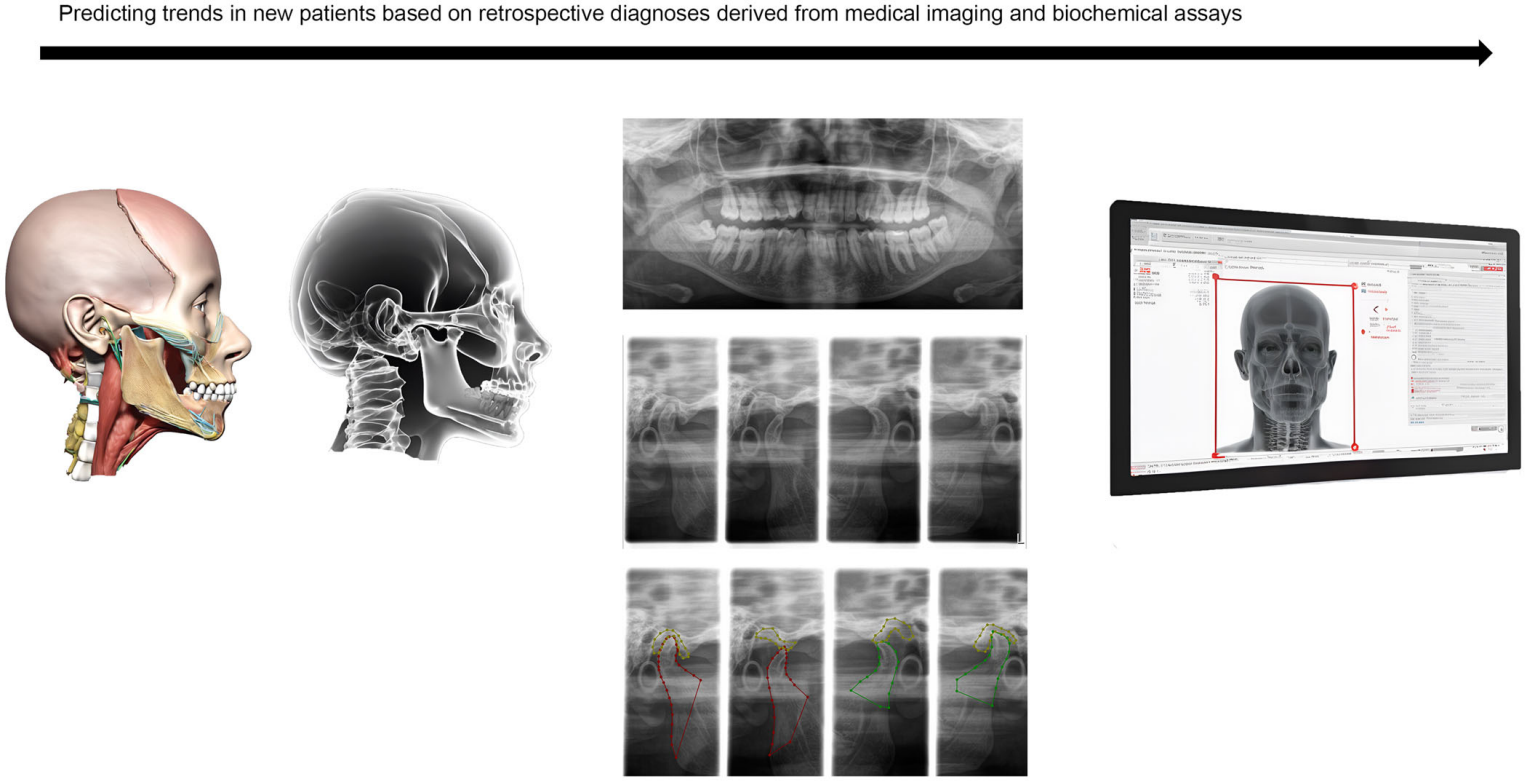
Predicting trends in condylar head morphology from imaging data.

AI‐guided interpretations of medical imaging, often considered a “black box,” pose challenges in understanding diagnostic decision rationale (Kim et al. 2021). Radiographic imaging, including panoramic X‐rays, CT scans, and MRI, is selectively performed, with standard reference for predictive modeling often established from routine 2D panoramic or CBCT imaging, especially for inflammatory joint diseases (Farook and Dudley 2023a; Ahmad and Schiffman 2016). Figure 7 demonstrates a simplified illustration of how imaging data are used for making predictions. This workflow can predict or support a practitioner's diagnostic speculation regarding anatomical variations, dentofacial malformations, or alterations influenced by medical factors seen in the patient's imaging data (Figure 4).

### 4.2 Barriers to AI‐Based Predictions

Even with globally available large data sets, image acquisition errors frequently result in discarding significant data. Radiomics‐related errors, such as poor patient positioning, inappropriate resolution, and exposure variations, often lead to data exclusion (Farook and Dudley 2023a; Langlais and Miller 2016). Patient position errors can distort teeth and mandible appearance during imaging (Langlais and Miller 2016). Power and exposure time variations may create lighter or darker images, impacting diagnostic accuracy. Transitioning traditional film images to digital platforms can introduce errors, and artifacts within CBCT scans may introduce challenges in evaluating condylar bone density in arthritis (Haghnegahdar et al. 2018; Tsai et al. 2020). MRI scans, considered the gold standard for diagnosing disc position, may introduce biases with operating parameter variations (Orhan et al. 2021; Nozawa et al. 2022). External factors can affect infrared imaging for masticatory muscle activity. Rarely do patients undergo all mentioned imaging modalities, potentially leading AI models to assume trends and patterns, even when successfully predicting outcomes (Shin 2021). While outcomes are often published and generally well received, current practices are emphasizing more on extracting explanations from the AI that guided its decision‐making (Arian et al. 2023; Xu et al. 2019).

### 4.3 Looking at Hard and Soft Tissue Together for Better Predictions

Prediction models based on the relationship between TMJ hard tissue and masticatory muscles serve as diagnostic tools, aiding in identifying and differentiating occlusal interferences and disc derangements (Lee, Jha, and Kim 2021). The highly individual trait of muscle function is influenced by social, environmental, and parafunctional habits that require a detailed history to cover. Muscle activity can now be quantified and normalized through deep learning to facilitate generalized comparisons across diverse populations (Barron et al. 2022; Farook, Haq, Ramees, and Dudley 2024a). These models shape personalized treatment plans, fostering transparency, patient comprehension, and compliance, offering evidence‐based reassurance, especially in conditions with potential spontaneous resolution (Warwick and Salkovskis 1985; Castaneda et al. 2015). Model‐guided differential diagnoses enhance communication among practitioners of different specialties, addressing historically low inter‐rater reliability (Stegenga 2010). Elevator and depressor muscle function assessment is crucial for evaluating TMJ conditions, with imbalances and parafunctional habits contributing to dysfunction (Farook, Haq, Ramees, and Dudley 2024a). Conditions affecting either the bony or muscular components exhibit shared symptoms that complicate a diagnosis (Stegenga 2010). Subtle signs such as jaw deviation during mouth opening are a visible manifestation of underlying TMJ or muscle issues and can be used to model predictions (Farook, Haq, Ramees, and Dudley 2024a; Troka et al. 2022). Using deep learning for data established from noninvasive electrognathography, electromyography, and 3D intraoral scans can establish robust quantitative relationships, particularly in normalizing muscle intensity to predict activities with low absolute error margins (Farook, Haq, Ramees, and Dudley 2024a).

### 4.4 Using Every Noninvasive Chairside Tool at Disposal to Predict Norms

**Figure 8:**
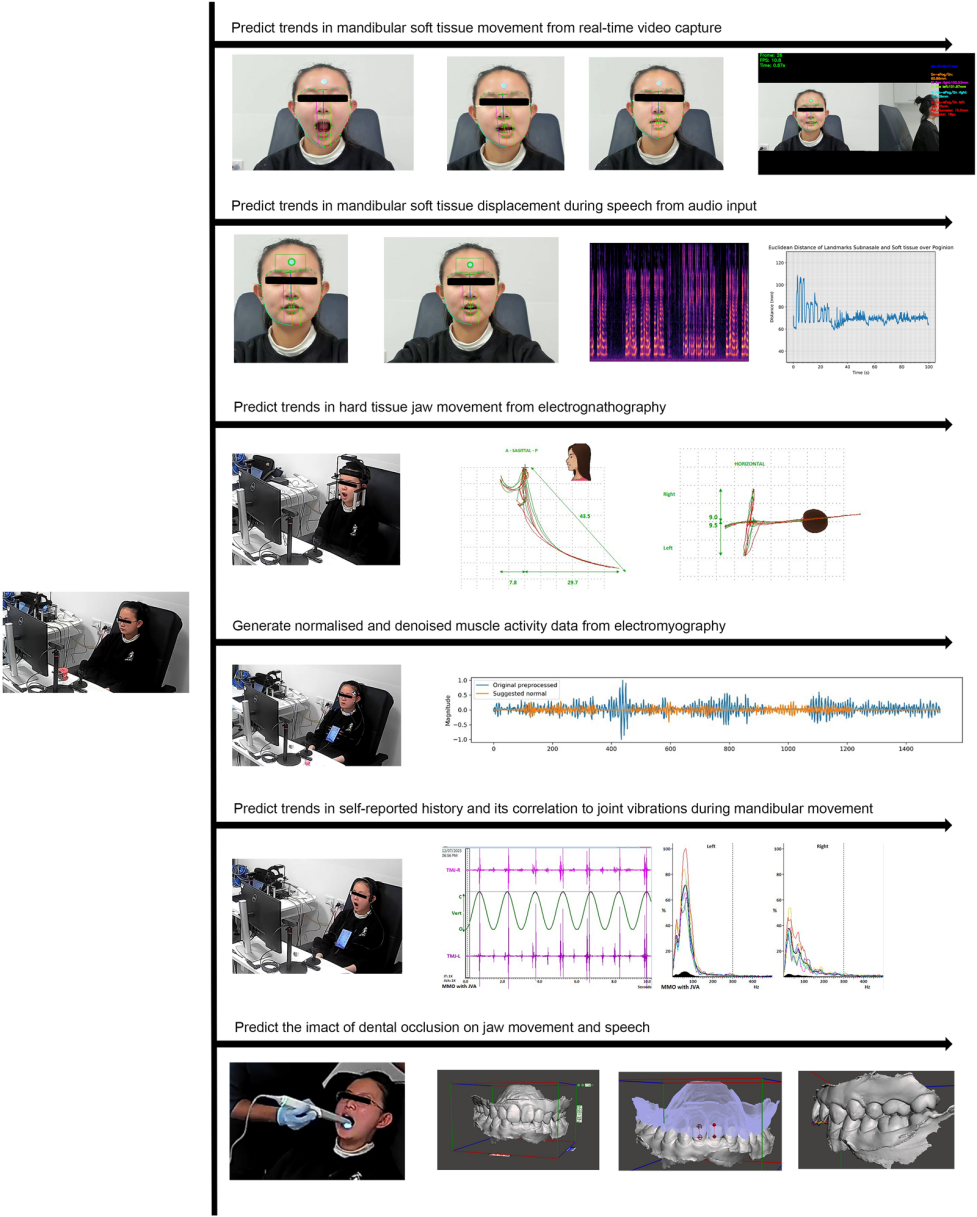
The array of noninvasive chairside tools that can be used to generate predictor data.

A decision support system, trained with substantial data sets can successfully predict the location, direction, and extent of atypical deviation in TMD, identifying common trends within demographics and guiding evidence‐based, tailored treatment plans. Figure 8 demonstrates the variety of tools used together to collect data for use as predictor variables, ultimately contributing to the achievable outcomes outlined in Figure 4.

## 5 Professional and Public Consensus

AI‐driven predictive modeling in dentistry stands as one of the fastest growing sub‐fields in healthcare research, outpacing the clinical translation of research findings (Schwendicke, Samek, and Krois 2020; Khanagar et al. 2021). Its swift evolution, deemed impossible just two decades ago, has already received multiple iterations in computer vision, a type of AI capable of detecting features from images, recordings, and 3D models; and natural language models, a form of AI that enables interaction with humanoid precision. As AI evolves from clinician‐controlled supervised deep learning to autonomous unsupervised learning, its diagnostic accuracy is reaching formidable levels, sparking debates about its acceptance and regulation in clinical dentistry (Dashti et al. 2024).

### 5.1 Research in Australia

**Figure 9:**
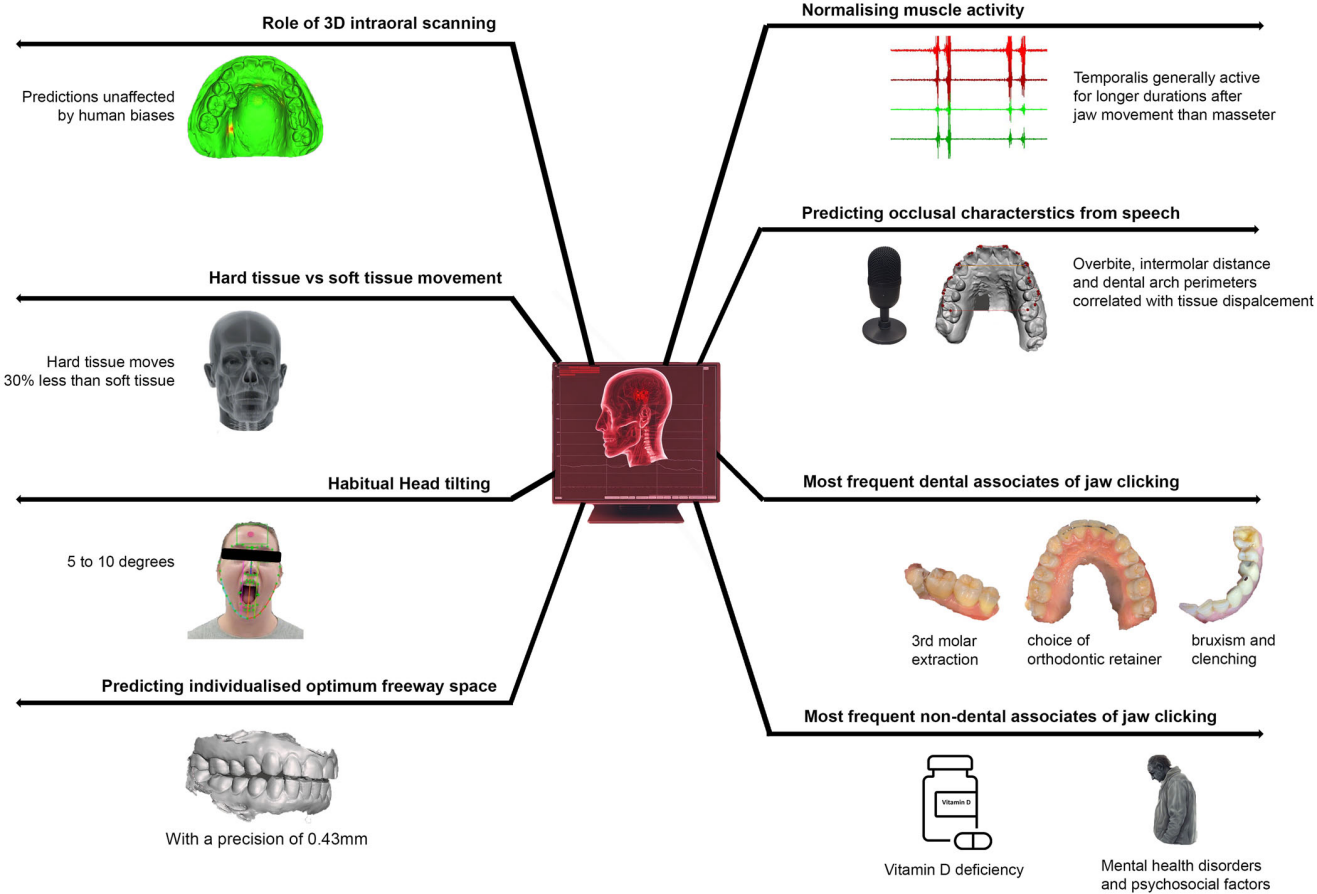
A summary of findings from investigations conducted by the authors in Australia.

An investigation into the impact of intraoral scanners and operator skills on 3D dental cast scans for predictive modeling revealed no significant effect on dimensional accuracy (Farook, Ahmed, Giri, Rashid, Hughes, and Dudley 2023). It was observed that such evaluations could be conducted using portable handheld computers with efficacies equivalent to larger workstations (Farook and Dudley 2024). Tracking facial soft tissue landmarks through a consumer‐grade camera showed that hard tissue electrognathographic movement was 30% less than soft tissue, often accompanied by a 5–10 degree head tilt (Saad et al. 2024). Factors contributing to self‐reported jaw clicking were identified, with predictors including third molar extraction, vitamin D deficiency, mental health conditions, and parafunctional clenching (Farook, Ramees, and Dudley 2024b). Validation of masticatory muscle activity extraction from EMG signal images was achieved (Farook, Haq, Ramees, and Dudley 2024a). Predictive modeling for jaw movement trends using deep learning applications indicated a promising avenue for establishing a quantitative relationship between hard and soft tissue components of the temporomandibular joint complex (Farook, Haq, Ramees, and Dudley 2024b). Additionally, factors predicting occlusal contributions to phonetics, such as variations in dental arch perimeters, presented outcomes in altered soft tissue displacement during phonetic articulation (Farook, Ramees, and Dudley 2024a). Finally, convolutional neural networks achieved a commendable precision of 0.43 mm in predicting freeway space from 121 unique jaw movement parameters (Farook, Haq, Ramees, and Dudley 2024c). The investigations conducted in South Australia explored predictive modeling in the fields of occlusion and TMJ functionality. Figure 9 illustrates the overall findings.

### 5.2 Community Engagement

In November 2023, the authors conducted community engagement sessions exploring opinions on the prevailing sentiments toward AI in dentistry. The feedback from young dentists, academics, and healthcare professionals reflected limited knowledge of the technology. However, they expressed acceptance and enthusiasm for implementing AI technologies in line with global trends in professional acceptance (Ranjana et al. 2021; Syed et al. 2023). They saw the potential to save time and expenses while enhancing evidence‐backed treatment plans for their patients. However, patients and members of the general community expressed reservations, perceiving AI as merely a decision support system rather than recognizing its potential to substitute for a lack of human dental professionals, especially in regions with limited dental care outreach (Alowais et al. 2023).

### 5.3 Community Perception

Like recent reports from other parts of the globe, South Australian community members based their perceptions on beliefs shaped by pop culture and harbored concerns about the potential misuse of AI (Zhan et al. 2023). The lack of awareness regarding the beneficial applications of AI, such as providing real‐time translation services, maintaining appointments and priority lists for patients, aiding in complex diagnoses, providing evidence‐based reassurance, and offering support while the patients await physical appointments, contributed to our observed public reluctance to share their data with AI systems. Those with expertise in computer science, statistics, and mathematics recognized the potential benefits of AI but had mixed feelings about entrusting their data to AI entities governed by corporations.

Surprisingly, cost did not emerge as a significant factor influencing decisions. Individuals expressed a willingness to pay full amounts and endure lengthy wait times to talk to human dental professionals, even when an ‘AI dentist’ that would draw from the collective experiences of thousands could theoretically offer preliminary recommendations at a moment's notice and at a level almost similar to human practitioners (Ye et al. 2024). This sentiment persisted despite knowing of the AI entity's ability to engage patients using methods similar to widely accepted language models such as ChatGPT (Li et al. 2024). These initial findings are some of the first of their kind in this field and certainly warrant further investigation.

## 6 Conclusion

This narrative review demonstrates the tangible impacts of predictive modeling and deep learning in identifying clinical trends related to occlusion and TMJ function. Further research is recommended to explore ways to enhance community acceptance of AI‐driven dentistry, emphasizing the role of dental professionals in educating patients about the potential benefits that such technology may bring in the future.

## Author Contributions

**Taseef Hasan Farook:** conceptualization, methodology, investigation, resources, writing–original draft, writing–review and editing. **James Dudley:** conceptualization, methodology, investigation, writing–review and editing, supervision, project administration, funding acquisition.

## Ethics Statement

The University of Adelaide Human Research and Ethics Committee approved research on predictive modeling of jaw movement and occlusion titled “The use of electronic and computerised tracking to determine trends in maxillomandibular trajectory, relation, and phono‐articulation” (HREC H‐2022‐185). All participants provided written consent before participating. Additionally, community engagement sessions were conducted to support a project titled “Artificial intelligence for restorative, endodontic, and surgical modelling of South Australian dental healthcare,” approved by the University of Adelaide Human Research and Ethics Committee (HREC H‐2023‐073).

## Data Availability

Data sharing is not applicable to this article as no data sets were generated or analyzed during the current study.
